# Xanthogranulomatous Salpingooophoritis: The Youngest Documented Case Report

**DOI:** 10.1155/2015/237250

**Published:** 2015-05-31

**Authors:** Harshawardhan Tanwar, Avinash Joshi, Vinayak Wagaskar, Siddharth Kini, Manoj Bachhav

**Affiliations:** ^1^Department of Urology, Seth G.S Medical College & KEM Hospital, Mumbai, Maharashtra, India; ^2^Department of Surgery, Government Medical College, Miraj, Maharashtra, India; ^3^Department of Plastic Surgery, Seth G.S Medical College & KEM Hospital, Mumbai, Maharashtra, India

## Abstract

*Background*. Xanthogranulomatous inflammation is an uncommon affection of the female genital tract. The youngest case reported of xanthogranulomatous salpingooophoritis in literature was by Shilpa et al. in 2013 in an eighteen-year-old female. *Case Report*. We report a case of 2-year-old female child with right-sided xanthogranulomatous salpingooophoritis presented as mass in abdomen. This is a case report of the youngest documented case of xanthogranulomatous salpingooophoritis in literature. As per abdominal examination, there was generalized distention of abdomen and a mass was palpable which was arising out of pelvis more on the right side. The ultrasonography (USG) abdomen and pelvis revealed a thick-walled mass measuring 9.2 cm × 6.0 cm × 7.6 cm in pelvis. We did right salpingooophorectomy of the patient. On histopathology, the diagnosis of xanthogranulomatous salpingooophoritis was confirmed. *Conclusion*. Clinical presentation, radiological appearance, and gross features of xanthogranulomatous lesions of ovary can mimic neoplastic lesions and lead to misdiagnosis. Though, it is very rare in pediatric age group, xanthogranulomatous salpingooophoritis as one of the differential diagnoses should be kept in mind while dealing with tuboovarian masses in this age group.

## 1. Introduction

Xanthogranulomatous inflammation is an uncommon cause of chronic destructive process of the normal tissue of the affected organ. It is characterized by accumulation of lipid-laden foamy macrophages (foamy histiocytes with small dark nuclei and clear cytoplasm) intermixed with lymphocytes, giant cells, and plasma cells [1]. Most common organ affected by xanthogranulomatous inflammation is kidney [2]. Other organs of xanthogranulomatous inflammation involvement are stomach, anorectal area, bone, urinary bladder, testis, and epididymis. Xanthogranulomatous inflammation of the female genital tract is rare and if found is mostly limited to the endometrium [3]. Only a few cases involving the ovary and fallopian tube have been reported [3, 4]. Clinical presentation, radiological appearance, and gross features of xanthogranulomatous lesions of ovary can mimic neoplastic lesions and lead to misdiagnosis. After extensive search of literature, we found that till date only 15 related cases of xanthogranulomatous oophoritis of fallopian tube or ovary have been reported in the literature, with average age of 31 years [5]. The youngest case reported of xanthogranulomatous salpingooophoritis in literature was by Shilpa et al. in 2013 in an eighteen-year-old female [6]. We report a case of 2-year-old female child with right-sided xanthogranulomatous salpingooophoritis presented as mass in abdomen. This is a case report of the youngest documented case of xanthogranulomatous salpingooophoritis in literature.

## 2. Case Report

**Figure 1:**
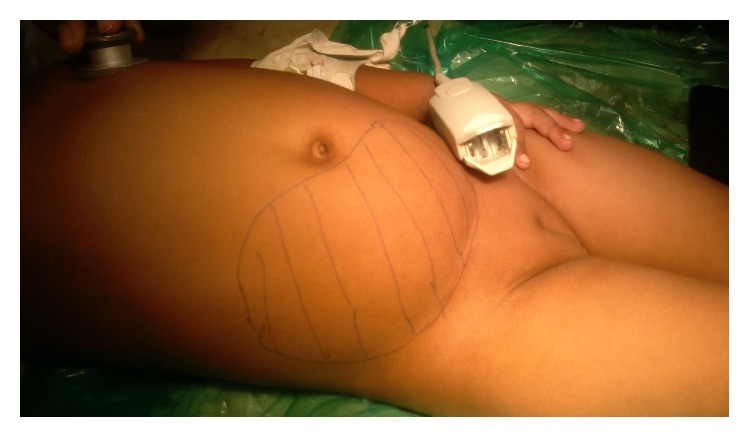
Clinical image with the outlining of the mass.

A 2-year-old female child presented with pain in abdomen with abdominal distention and fever since 8 days, as per the history narrated by her mother. She also had history of nausea, vomiting (nonbilious), and constipation since 2 days. As per abdominal examination, there was a generalized distention of abdomen and a mass was palpable which was arising out of pelvis more on the right side. The mass was extending from the right iliac region up to just below umbilicus and crossing the midline towards the left side in the suprapubic region (Figure 1). Mass on palpation was of mixed consistency, tender with no localized increase in temperature, and a size of 10 cm × 12 cm approximately. There were no signs of peritonitis on palpation.

**Figure 2:**
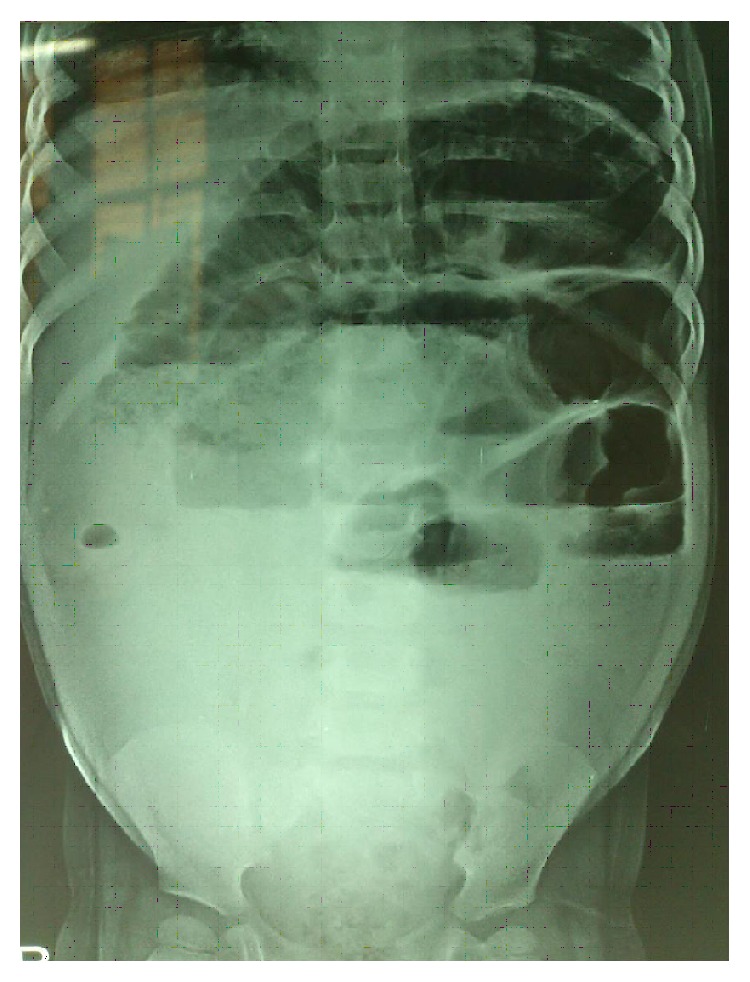
X-ray erect abdomen showing air fluid levels.

The ultrasonography (USG) abdomen and pelvis revealed a thick-walled mass measuring 9.2 cm × 6.0 cm × 7.6 cm in the pelvis. The right ovary was not seen separately from the mass and it showed irregular solid areas with large cystic changes and thin strands inside. The uterus and left ovary were normal. There was no free fluid in abdomen. Computed tomography (CT) scan showed a large heterogeneous mass arising from of right ovary. X-ray erect abdomen showed air fluid levels (Figure 2).

**Figure 3:**
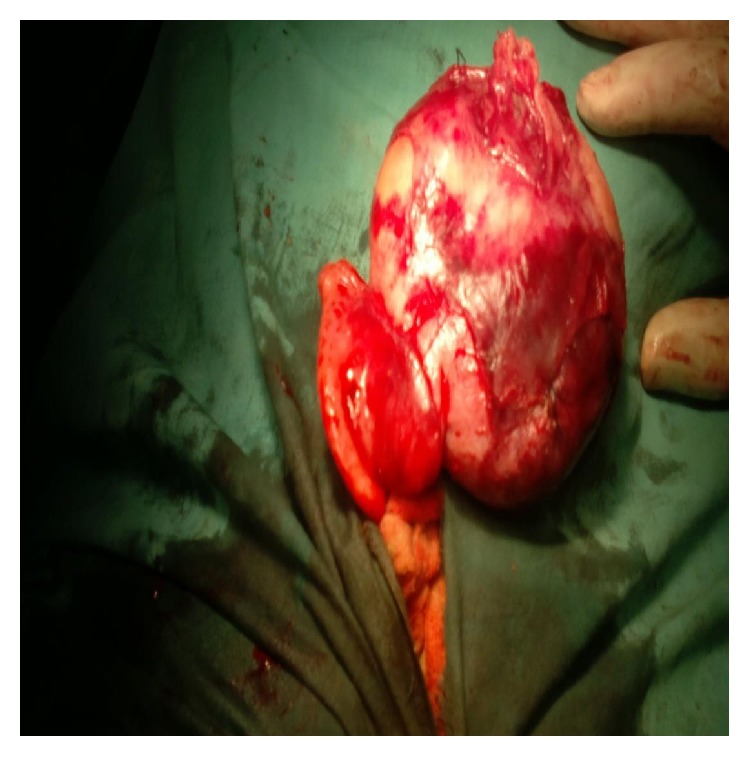
Exploratory laparotomy showing right tuboovarian mass.

**Figure 4:**
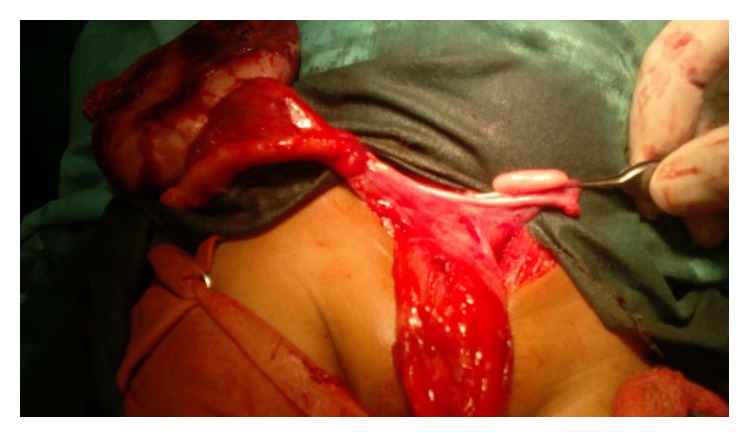
Right tuboovarian mass and normal left ovary and fallopian tube.

Hematological investigation showed a hemoglobin of 6.0 gm%, total leucocyte count of 12,000/mm^3^ with polymorphs 80%, lymphocytes of 20% and platelet count of 2 lakhs/mm^3^, and ESR 26 mm/hr, serum creatinine was 0.7 mg%, liver function test, urine routine, and microscopy were within normal limits, and urine culture showed no organism. Alpha-fetoprotein value was 11.45 ng/mL (normal value: 0–15 ng/mL). Other tumor markers like CA 125 and CEA were also within normal limits. On exploratory laparotomy, we found a right tuboovarian mass of size approximately 10 cm × 6 cm × 5 cm with distended small bowel loops and no ascites or pus in the abdominal cavity (Figure 3). We did right salpingooophorectomy of the patient and specimen was sent for histopathology reporting (Figure 4).

**Figure 5:**
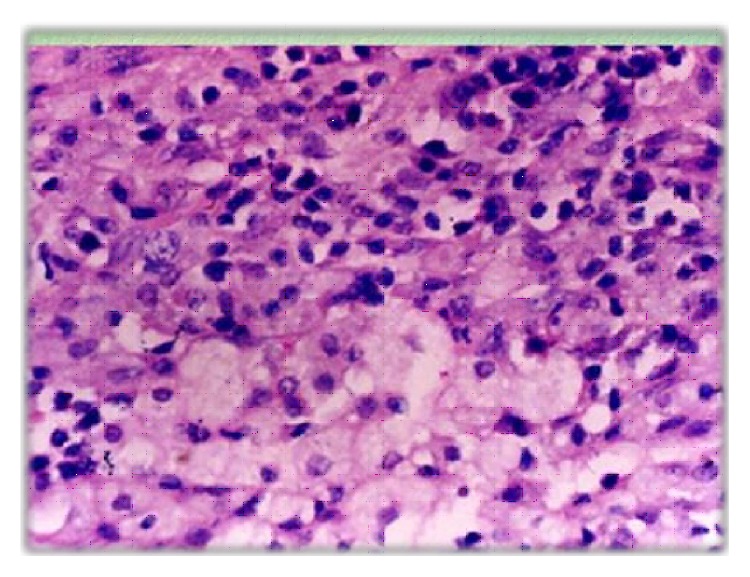
Histopathology slide of the specimen showing foamy histiocytes in higher power view.

On histopathology report, the specimen of the mass was yellowish in color grossly, had smooth outer surface, and measured 9 cm × 6 cm × 5 cm and the fallopian tube of the right side measured 2 cm in diameter and 4 cm in length with exudates on its surface and serous fluid in its lumen. The cut section showed multiple solid and cystic areas with small patchy areas of necrosis too. There was diffuse and dense infiltration of ovarian tissue by sheets of foamy histiocytes, lymphocytes, plasma cells, and polymorphs on microscopic examination. Microscopic examination also showed multiple microabscesses with extensive areas of hemorrhagic and infarctoid necrosis, with areas of marked fibrosis (Figure 5). The specimen tissue was subjected to special stains like Acid fast stain, PAS, and GMNS stain and was negative for microorganisms. Based on these unique and characteristic histopathology features, the diagnosis of xanthogranulomatous salpingooophoritis was confirmed. Postoperatively, patient was given intravenous antibiotics (3rd generation Cephalosporins) for 5 days. Patient made good and uneventful recovery postoperatively and was discharged on the 7th postoperative day.

## 3. Discussion

Xanthogranulomatous inflammation of the female genital tract is very rare and if involved is confined to the endometrium. However, there are documented cases of involvement of vagina, cervix, fallopian tube, and ovary. The youngest documented case is of 18-year-old female by Shilpa et al. [6]. Kunakemakorn et al. described the first case of xanthogranulomatous inflammation of serosa of uterus, left fallopian tube, and ovary in his report of inflammatory pseudotumor in the pelvis in 1976 [7]. Till date, only 15 cases of xanthogranulomatous inflammation of the female genital tract are reported in literature out of which there are 7 cases with unilateral ovary, 5 cases with unilateral fallopian tube, 1 case with bilateral fallopian tube involvement, and only 2 cases with ovary and fallopian tube showing simultaneous involvement [8]. The pathogenesis of xanthogranulomatous oophoritis is unclear and many theories that are of etiopathogenesis have been postulated, such as theory of infection, endometriosis, intrauterine contraceptive device, inborn errors of lipid metabolism, and drug induced. Amongst these theories, the most accepted theory is of infection, which is supported by clinical evidence of infection and growth of bacteria such as* Escherichia coli*,* Bacteroides fragilis*, and* Proteus vulgaris* from the affected tissue by culture [4]. The cases associated with endometriosis are often termed as pseudoxanthomatous salpingitis (PXS). Some authors think that PXS and xanthogranulomatous salpingitis are not the same entity [9, 10]. For Furuya et al., PXS appears as a xanthogranulomatous inflammation secondary to endometriosis [9]. On the other hand, they suggested that pelvic inflammatory disease associated with xanthogranulomatous changes should be termed as a pure XS [9]. Histologic and histochemical studies show that the histiocytic components of PXS present some differences with those of XS [9, 10]. Radiological findings of xanthogranulomatous may mimic malignant ovarian neoplasm and may lead to misdiagnosis. The clinical presentations of xanthogranulomatous oophoritis include fever, abdominal mass, and pain in the abdomen, menorrhagia, anemia, and anorexia. Gynecological examination reveals adnexal mass with tenderness with blood investigations showing elevated ESR and raised white blood cell count. Grossly, the affected ovary is enlarged with varying size and is like a tumor with yellowish appearance, with cystic areas within due to necrosis.

Differential diagnosis of xanthogranulomatous oophoritis includes infections like tuberculosis, fungal infections which can be ruled out by culture and special stains for the causative organisms. Malakoplakia is also one of the differential diagnoses. Wather presumed that malakoplakia and xanthogranulomatous inflammation were identical chronic inflammatory diseases [11]. In malakoplakia, the basophilic cytoplasmic concentric calcific bodies within histiocytes (Michaelis-Gutmann bodies) are found which were absent xanthogranulomatous inflammation ruling out this condition. Neoplastic lesions should be ruled out with detailed investigations as xanthogranulomatous oophoritis can be easily confused with ovarian malignancy, clinically, radiologically, and pathologically. Immunohistochemistry helps in confirming the diagnosis included, but it is seldom required in the presence of characteristics histopathological features. Treatment of choice for xanthogranulomatous salpingooophoritis is salpingooophorectomy as done in this case. Antibiotic therapy has been attempted, but it has not succeeded in reducing ovarian mass [7].

## 4. Conclusion

Though it is very rare in pediatric age group, xanthogranulomatous salpingooophoritis as one of the differential diagnoses should be kept in mind while dealing with tuboovarian masses in this age group. This will help in preventing misdiagnosis and radical surgeries if misdiagnosed as neoplasm of ovary. In a proven case of xanthogranulomatous salpingooophoritis, investigations should be done to find the causative factors, such as immunodeficiency disorders, diabetes, inborn errors of lipid metabolism, and pelvic inflammatory disease.
